# Cardiovascular disease detection: A hybrid machine learning-AI framework for personalized diagnosis and risk assessment

**DOI:** 10.1371/journal.pone.0335421

**Published:** 2025-10-29

**Authors:** Medhat A. Tawfeek, Ibrahim Alrashdi, Madallah Alruwaili, Hisham Allahem

**Affiliations:** 1 Department of Computer Science, College of Computer and Information Sciences, Jouf University, Sakaka Aljouf, 72388, Saudi Arabia; 2 Department of Computer Engineering and Networks, College of Computer and Information Sciences, Jouf University, Sakaka Aljouf, 72388, Saudi Arabia; 3 Department of Information Systems, College of Computer and Information Sciences, Jouf University, Sakaka Aljouf, 72388, Saudi Arabia; Deakin University, AUSTRALIA

## Abstract

Cardiovascular disease (CVD) is considered the number one killer disease in the world, underlining the importance of the application of more accurate diagnostic and therapeutic tools. Traditional screening procedures usually do not provide identification and guidance based on individual peculiarities that might result in less than beneficial results. This study seeks to create a hybrid computational framework that synergistically integrates a Support Vector Machine (SVM) classifier, a Particle Swarm Optimization (PSO) algorithm for hyperparameter tuning, and an AI-based interpretation module (SHapley Additive exPlanations, SHAP) to enable early diagnosis and risk assessment beyond various profiling of patients. A mathematical model was developed to provide the framework to deal with the diagnostic complexity of cardiovascular disease. Machine learning (ML) and AI techniques are then used to improve clinical decision-making. The proposed framework employs a variety of forms of patient data, namely electronic health records, medical images, and genomic data, to construct patient models. It utilizes advanced algorithms to enable accurate disease prognosis, identify high-risk individuals for early intervention, and facilitate personalized treatment strategies. This approach will help to eliminate the expense of ineffective therapies, shorten delays in care, and eventually improve patient outcomes and quality of life. Preliminary results on the MIMIC-III clinical database (v1.4) showed that the proposed framework performs better than previous methods by achieving higher accuracy 98.4%, precision 97.5%, recall 96.4%, F1 score 96.9%, and AUC-ROC 97.35%. Moreover, the sensitivity 96.4%, specificity 98.7%, and a low negative likelihood ratio (0.036) of the proposed framework demonstrate its ability and power in identifying high- and low-risk patients. The hybrid ML-AI framework provides an improved way for early detection of cardiovascular disease, which helps in personalizing treatments for patients. It also enables healthcare delivery through its combined predictive power to improve healthcare service.

## Introduction

CVD has been identified as a major global health threat, and mortality rates from this disease continue to rise despite better knowledge, evidence, and technology [1]. In fact, CVD is the leading cause of death, with 18.6 million deaths annually, and the World Health Organization states that CVD is diverse and complex disease [2,3]. As the challenges of diagnosing and treating heart disease continue, they drive the need for precise solutions [4,5]. Traditional medical methods do not successfully handle the various connections between genes, life experiences, and health habits that create CVD problems [6]. Despite significant advances in medical technologies, disparities in healthcare delivery, rising costs, and an aging population pose significant barriers to effective care [7]. The nature of heart disease due to multiple factors, including genetics, lifestyle, and environment, makes it difficult to solve through conventional technologies. This persistent disparity calls for new, actionable approaches that can address other aspects of care delivery that vary by patient characteristics, thereby revolutionizing the management of cardiovascular disease. AI and ML are creating powerful new options to solve healthcare problems. The need for specific, high-quality solutions has emerged as a necessity, especially as global healthcare providers struggle to contain an aging population and rising healthcare costs [8]. This is where AI and ML-based personalized medicine comes in as an efficient solution to this problem and allows healthcare professionals to take an individualized, data-driven approach to tackling heart disease [9,10]. Recent studies validate the value of ML and deep learning in CVD prediction [11–22]. Using suspected Coronary Artery Disease (CAD) patient data, researchers investigated whether there are ways of employing ML to estimate all-cause mortality [11]. The study in [12] presents a narrative of wearable sensors in remote health monitoring inclusive of types/uses and challenges. The quality of ML approaches was examined in improving the reliability of heart failure mortality prediction and uncovering clinically relevant subgroups of heart failure patients [13]. The role of providing fairness and bias in ML in healthcare was proposed in [14] with the promotion of equality in the implementation of AI. The study in [15] tries to describe the clinical usability of a non-invasive wearable device that has a Micro Electro Mechanical systems (MEMS) pressure sensor array, which is used to analyze the arterial pulse waveform and heart rate in addition to atrial fibrillation. A model for cardiovascular disease prediction using a decision tree and naive Bayes model was proposed in [16], with an effort to facilitate accurate assessment of heart disease risk. A deep learning neural network is used in [17] to diagnose heart disease and, thus, show how the model can detect features and patterns within medical data. Useful applications of the K-nearest neighbor (KNN) algorithm, especially the prediction of heart diseases, are discussed [18]. A significant smart healthcare architecture that incorporates both deep learning and ML for rapid diagnosis of heart diseases has been developed in [19]. New to the field of data classification, SVM remain relevant as a classification technique, essential in concrete tasks such as inferring heart diseases by constructing a hyperparameter that gives the maximum of known divisive classes in the feature space. This approach gives a fairly good result, especially in the case of multidimensional data [20]. The authors of [20] aimed to predict heart disease using two ML techniques: SVM and artificial neural network (ANN). For analyzing the link between cardiovascular disease and multiple risk factors, the logistic regression method was applied [21]. The application of AI in the diagnosis and treatment of cardiovascular disease was examined in [4], and it was emphasized how AI models enhance accuracy. The authors in [22] proposed the use of a Convolutional Neural Network (CNN) model in helping to enhance the correct rate of a heart disease diagnosis. For a better understanding of the related work presented above, Table 1 provides a comparative summary of major studies on CVD using ML and deep learning. The table contrasts their methods, datasets, performance measures, and key findings.

**Table 1:** A comparative summary of relevant work on cardiovascular disease prediction using machine learning and deep learning. The table compares methods, datasets, key performance metrics, and key findings. This review highlights the major issues in the area, such as the interpretability, the generalizability, the cost of computation, and the privacy of data, all of which motivate the proposed hybrid model.

Ref	Method	Dataset	Performance Measures	Key Findings
[11]	ML Predictive Model for CAD Mortality	collected from multiple centers	sensitivity, specificity, AUC	lack of generalizability due to its focus on a specific patient population and Interpretability Issues.
[12]	Wearable Sensor Monitoring	Collected by wearable devices	accuracy, battery life, data security, and user compliance	data privacy, security risks, short battery life of wearable devices.
[13]	ML-Based Heart Failure Mortality Prediction	dataset of heart failure patients not explicitly stated	accuracy, precision, recall, or F1-score	Limited Generalizability, Complex Implementation, Interpretability Issues.
[14]	Fair and Ethical ML in Healthcare	No specific dataset; focuses on theoretical and ethical issues	Not applicable; the study discusses ethical implications.	Risk of algorithmic bias, data privacy concerns, reduced transparency.
[15]	MEMS Wearable Monitoring	from individuals wearing	Accuracy of pulse waveform readings, heart rate measurement accuracy	Limited accuracy with motion, less effectiveness with low pulse amplitude.
[16]	DT-NB CVD Prediction	Patient records from current medical imaging	Accuracy, Sensitivity	Limited scalability, Potential overfitting with complex data.
[17]	Deep learning CVD Prediction	Publicly available heart disease dataset.	Accuracy and diagnostic performance	Risk of over-fitting, High computational resource requirements, Poor interpretability.
[18]	KNN Heart Disease Prediction	Medical data with features like age, cholesterol and others	Accuracy, precision, and recall.	Sensitive to the choice of ‘k’ value, high computational cost with large datasets,
[19]	deep learning/ML Heart Disease	Comprising patient medical records	accuracy, precision, recall, and F1-score	May introduce bias, Patient privacy, limited scalability across various healthcare settings.
[20]	SVM/ANN Heart Disease	Heart disease dataset	Accuracy, precision, recall	high computational complexity for ANN
[21]	Logistic Regression CVD	patient health records	ccuracy, precision, recall, and F1-score.	Limited generalizability, may fail to capture complex, nonlinear relationships between factors.
[4]	AI Cardio Support	data commonly used in cardiovascular AI applications	(AUC-ROC)	High costs and dependence on advanced infrastructure, interpretability issues
[22]	CNN TL Heart Disease	datasets that included patient health records	accuracy, precision, recall, and F1-score	potential for overfitting and specific datasets may restrict the applicability, Risk of Overfitting.

Current models face challenges such as interpretability and generalizability, computational requirements, and ethical concerns. Some rely on nonspecific structures, are sensitive to feature selection, or lack the ability to adapt to diverse healthcare settings. These limitations often result in inadequate targeting of cardiovascular disease complexities.

The proposed hybrid framework—a term we use to describe the tight integration of an optimized classifier (SVM), a meta-heuristic optimizer (PSO), and an interpretability component (SHAP)—is designed to address these gaps by providing a solution that is accurate, computationally efficient, and clinically interpretable. Compared to the previous approaches, our framework has the following major improvements:

- The proposed framework incorporates dynamic and real-time risk adaptation, allowing patient risk scores to be continuously updated through the integration of electronic health record streams and wearable device inputs.
- The framework also incorporates flexibility, determining the weighting of various data patterns when some inputs are missing or incomplete. This flexibility, supported by the dynamic weighting system, enables the model to achieve efficient performance even when exposed to a realistic environment where genomic or other data sources are not always available.
- This integration improves the accuracy of cardiology based on the patient profile. The proposed method goes beyond static methods of prediction strategies, and instead enhances timely clinical decision-making by responding to patient data as it evolves.
- Interpretability-Optimization Fusion: It incorporates explainability in the optimization mechanism.

Moreover, this study involves presenting a formal mathematical model for cardiovascular disease detection, incorporating data integration techniques to build and validate a clinically relevant, interpretable risk assessment framework.

The remainder of this paper is divided into the following sections: Section 2 moves on to present a mathematical model to formulate the CVD problem and outlines strategies for seamlessly integrating diverse data sources. Section 3 delves into the design of novel AI/ML models for accurate personalized risk prediction. This section will outline the specific algorithms used. Section 4 provides comprehensive analytical results and comparisons of established approaches in the field of CVD. Section 5 presents a detailed discussion of the experimental results and examines the implications for real-world applications. Section 6 provides the conclusion and outlines future work.

## Problem formulation: CVD risk assessment and personalized treatment

The formulated model combines several patient-centric data inputs to improve the accuracy of patient data and take into account their specific needs. The dynamic risk assessment model consists of four input vectors, including medical images, clinical tests, genomic information, and the patient’s medical history. These data are used to predict patient risks, group similar patients, and help improve treatment strategies. The model is improved by adding a loss function to train the system, which helps overcome bias and provides personalized results. The problem is expressed in terms of four important input vectors, a decision function is defined, and an appropriate loss function is used.

### Input vectors for risk prediction

The four key input vectors are defined as V_1_, V_2_, V_3_, and V_4_, each of which represents different features of a patient’s data. These vectors are actually fed to a model that can predict the probability of cardiovascular events and the treatment strategy.

- Vector V_1_ (Medical Imaging Data) includes features obtained from some medical imaging modalities such as echocardiogram or other modalities such as MRI or CT scan. Equation (1) represents this vector. It is used in the analysis of the structural and functional changes in the heart, including hypertrophy or stenosis of the left ventricle.

$${\text{V}}_{\text{1}}\;=\;[{\text{I}}_{\text{1}},{\text{I}}_{\text{2}}\;,\ldots ,\;{\text{I}}_{\text{m}}\;]$$ (1)

Where, I_f_ is individual features of imaging and m is the count of features extracted.

- Vector V_2_ (Clinical Test Results) contains blood pressure, lipids, pulse rates and oxygen saturation measurements that represent the patient’s current physiological condition. It is given by Equation (2)

$${\text{V}}_{\text{2}}\;=\;[{\text{T}}_{\text{1}},\;{\text{T}}_{\text{2}},\;\ldots ,\;{\text{T}}_{\text{k}}]$$ (2)

Where, T_s_ is the individual clinical test results, and *k* is the number of clinical feature taken into consideration.

- Vector V_3_ (genomic information) integrates annotations of genome and genetic data into an easy-to-read report about genetic risks for certain types of CAD. This integration makes it possible to achieve a better comprehension of how the genetic endowment and other genomic features influence cardiovascular health. Equation (3) represents the vector of the genomic information.

$${\text{V}}_{\text{3}}\;=\;[{\text{G}}_{\text{1}},\;{\text{G}}_{\text{2}},\ldots ,\;{\text{G}}_{\text{n}}]$$ (3)

Where, G_j_ encapsulates genomic data including whole genome sequence information and genetic modifications that may influence gene expression and thus patient risk, or represents specific genetic markers (such as single nucleotide polymorphisms, or Single Nucleotide Polymorphism (SNPs)) known to be associated with increased risk of cardiovascular disease, *n* is the number of markers.

- Vector V_4_ (Medical History) includes the patient’s medical history, like previous diagnosis and treatment, surgical history, and disease history, and is represented by Equation (4).

$${\text{V}}_{\text{4}}\;=\;[{\text{H}}_{\text{1}},\;{\text{H}}_{\text{2}},\ldots ,\;{\text{ H}}_{\text{w}}]$$ (4)

Where, H_r_ refers to a history and progression-oriented factor, q is the total number of histories considered.

### Dynamic risk scoring and prediction

To predict the patient’s cardiovascular risk, we define a risk score R(t) at time t, which is dynamically updated as new data is acquired. This risk score is based on a function *F*, which takes the four input vectors as arguments as in Equation (5).


$$\text{R}(\text{t})=\text{ F}(\beta_{\text{1}}.{\text{V}}_{\text{1}}(\text{t})+\;\beta_{\text{2}}.{\text{V}}_{\text{2}}(\text{t})+\;\beta_{\text{3}}.{\text{V}}_{\text{3}}(\text{t})+\;\beta_{\text{4}}.{\text{V}}_{\text{4}}(\text{t}))$$ (5)

Where, F is explained below, which will be the result from the ML model for processing the time series data. The parameters βi serve the purpose of modifying the contribution of each vector in the overall prediction calculation. The model equates a new amount of data with an updated risk score R(t) in order to capture the dynamics of patient care in near real time.

### Decision function

The objective of the model is to classify a patient as high risk, where he or she needs to go through a bypass surgery, or as a low-risk patient who should be prescribed certain medications. The decision function F(X) is what is used to classify the kind of patients, and depending on the score that F(X) will provide, an individual will be said to be having heart disease or not. The decision function is computed by Equation (6).


$${\text{F}(\text{X})=\text{w}}^{\text{T}}\text{X}+\text{b}$$ (6)

Where, F(X) is the decision function that separates patients with high risk (positive category) from patients with low risk (negative category), *w* is the weight vector used to give different features different importance in the input vectors, and b is the bias term. As for the proposed model, its purpose is to find the appropriate weight vector *w* and bias factor *b* that allow the decision function to correctly classify patients, according to their individual personality criteria.

### Squared hinge loss function

The squared hinge loss function is specifically designed for classifiers such as SVMs, and it offers key advantages, especially when combined with AI and optimization algorithms such as PSO. It reduces the chance of obtaining a local minimum because it helps the model converge more effectively. The squared hinge loss for the binary classification problem is defined mathematically by Equation (7).


$$\begin{array}{c} L=\frac{\text{1}}{n}\sum {\mathrm{\backslash nolimits}}_{i=\text{1}}^{n}max(\text{0},\text{1}-{\text{Y}}_{i}\text{F}({\text{X}}_{i}){)}^{\text{2}} \end{array}$$ (7)

Where, L is the squared hinge loss function, n is the number of training samples, X_i_ is the feature vector of the sample number *i* of training data, Y_i_ is the true label (1 for patients with a high risk of heart disease and −1 for low risk), and F(X_i_) is also the decision function of the ML that calculates the ranking score of the classifier toward input X_i_

The loss function tries to punish predictions that are off the margin Y_i_ F(X_i_) ≥ 1; however, different from the basic Hinge Loss, the punishment level rises to the second power for the misclassified or margin-violating sample.

### Patient clustering

To improve risk assessment and treatment planning, patients are classified into groups based on the similarity of input vectors. Suppose *C_1_*, *C_2_*, …, *C_g_* are patient groups where *g* denotes the number of groups. Patients are grouped into subgroups based on their input vectors. These groups are formed using unsupervised learning algorithms. By scanning selected patients’ medical images, clinical tests, genetic markers, and medical records, patients are divided into groups with similar characteristics. Clustering can be formally defined by Equation (8).


$$\begin{array}{c} {\text{C}}_{\text{i}}=\{{\text{V}}_{\text{1}}(\text{i}),\;{\text{V}}_{\text{2}}(\text{i}),\;{\text{V}}_{\text{3}}(\text{i}),\;{\text{V}}_{\text{4}}(\text{i})|\text{similar metric}\} \end{array}$$ (8)

This clustering helps identify risk and disease treatment patterns in groups that share similar characteristics. In this way, each *C_i_* group can be linked to treatments that have been identified as effective for patients with similar characteristics and can be linked to specific treatment strategies that have been shown to be effective for patients with similar profiles.

### Optimization for personalized treatment

Once the dynamic risk score R(t) for the patient is determined, the next step is to find the treatment that minimizes the post-treatment risk given the patient’s limitations, including drug tolerance or the results of his or her previous treatments. Let Z_j_∈{0,1} be a binary decision variable. Z_j_ = 1 if treatment *j* is chosen; Z_j_ = 0 otherwise. The optimization is to reduce the expected future risk R(t + 1) with constraints such as budget constraint, or medical rules.


$$min\;E[R(t+\text{1})\;|\;Z_{j}]$$ (9)

subject to:


$$\begin{array}{c} \sum {\mathrm{\backslash nolimits}}_{\text{j}\in \text{T}}\text{Costj}.\text{ Zj }\leq \text{B} \end{array}$$ (10)

where Cost_j_ is the cost of treatment *j*, and *B* is the total budget.

This optimization guarantees that the decided treatment reduces future cardiovascular risk to a level that is acceptable and achievable within patient constraints.

The above formulation states that the proposed dynamic risk scoring model will combine different sources of information to generate an in-depth estimate of a patient’s cardiovascular risk.

## A hybrid SVM-PSO-AI framework for interpretable cardiovascular risk prediction

This section describes the hybrid model methodology with a modified SVM for classification, a modified PSO algorithm for SVM parameter optimization, and an AI module for model interpretation using SHAP values. The term “hybrid” here refers to the complementary fusion of these different methods into a single, common pipeline, rather than a set of predictive models.

**Fig 1:**
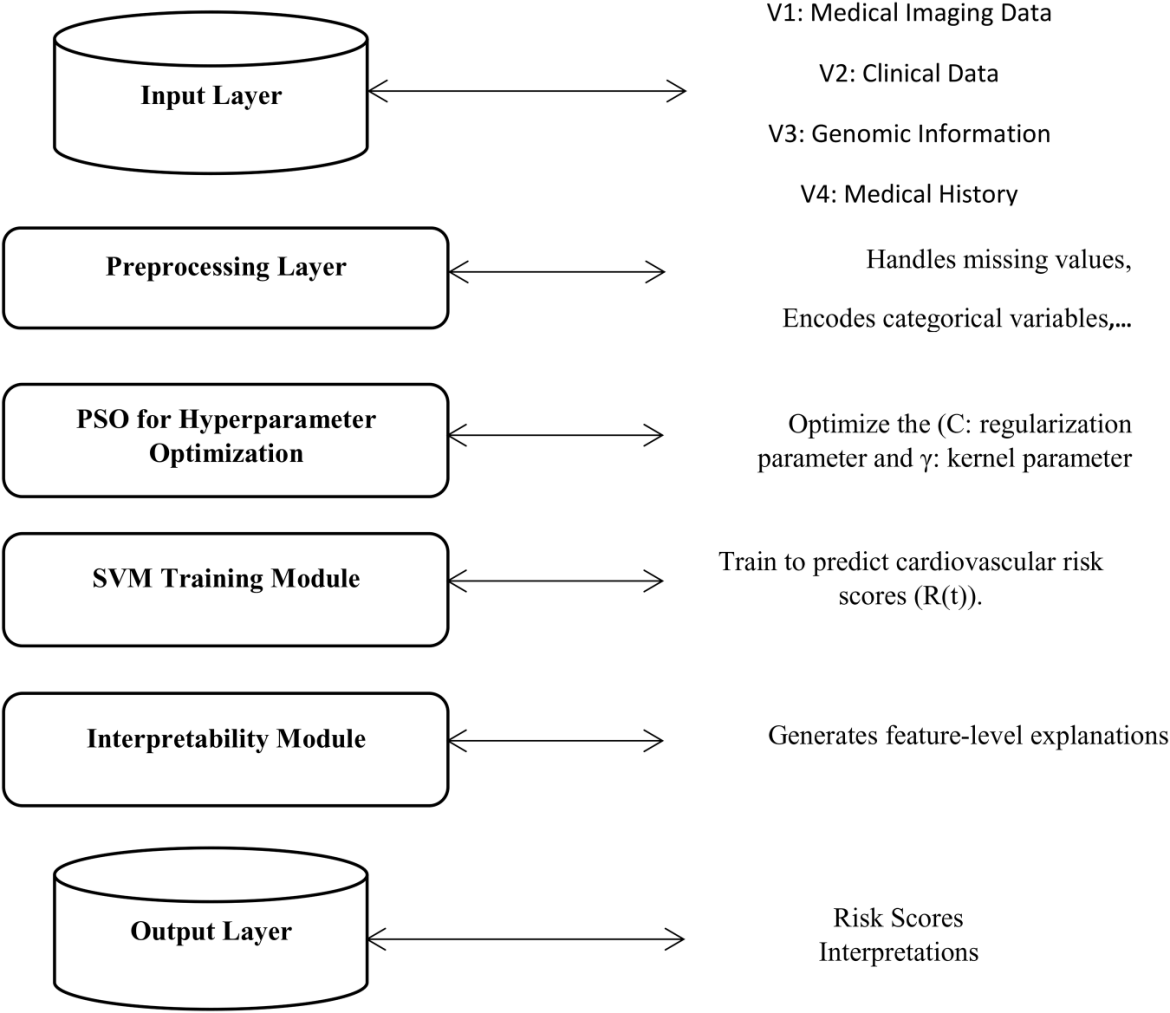
Schematic architecture of the proposed hybrid SVM-PSO-AI framework for cardiovascular risk prediction: It has four input vectors V1 (Medical Imaging), V2 (Clinical Data), V3 (Genomic Information) and V4 (Medical History). Data undergoes preprocessing before being processed by the core model. The Particle Swarm Optimization (PSO) algorithm optimizes the hyperparameters (C, γ) of the Support Vector Machine (SVM) classifier. The trained SVM generates a cardiovascular risk score. The obtained prediction is then interpreted by the SHapley Additive exPlanations (SHAP) module to provide feature-level explanations.

The proposed approach also resolves the current problem of considering the three techniques to accurately classify and estimate cardiovascular risks while optimizing model parameters and targeting dynamic and real-time risk assessments. The architecture in Fig 1 visually illustrates the integration of the components of the proposed hybrid model. As can be seen from Fig 1, the proposed hybrid model architecture integrates multiple components to provide a comprehensive framework for predicting and interpreting cardiovascular disease. The methodology consists of four key components that overview the hybrid model:

1. Preprocessing: Handling missing values, normalizing numerical features, encoding categorical variables, and dynamic class weighting is used to counteract the effects of class imbalance. This is done to maintain the quality and stability of data prior to the classification.
2. Modified SVM: Used as the classifier to give the probability estimates for cardiovascular risk assessment from patient background.
3. Modified PSO: Towards providing real-time reusability of the parameters of the SVM, such as the regularization parameter *C* and the kernel parameters for optimization.
4. AI Module: This adds an upper level of interpretation such that the contributions of each feature (example genetic markers or clinical history) can be formed and offer the required level of decision support in health care settings.

**Fig 2:**
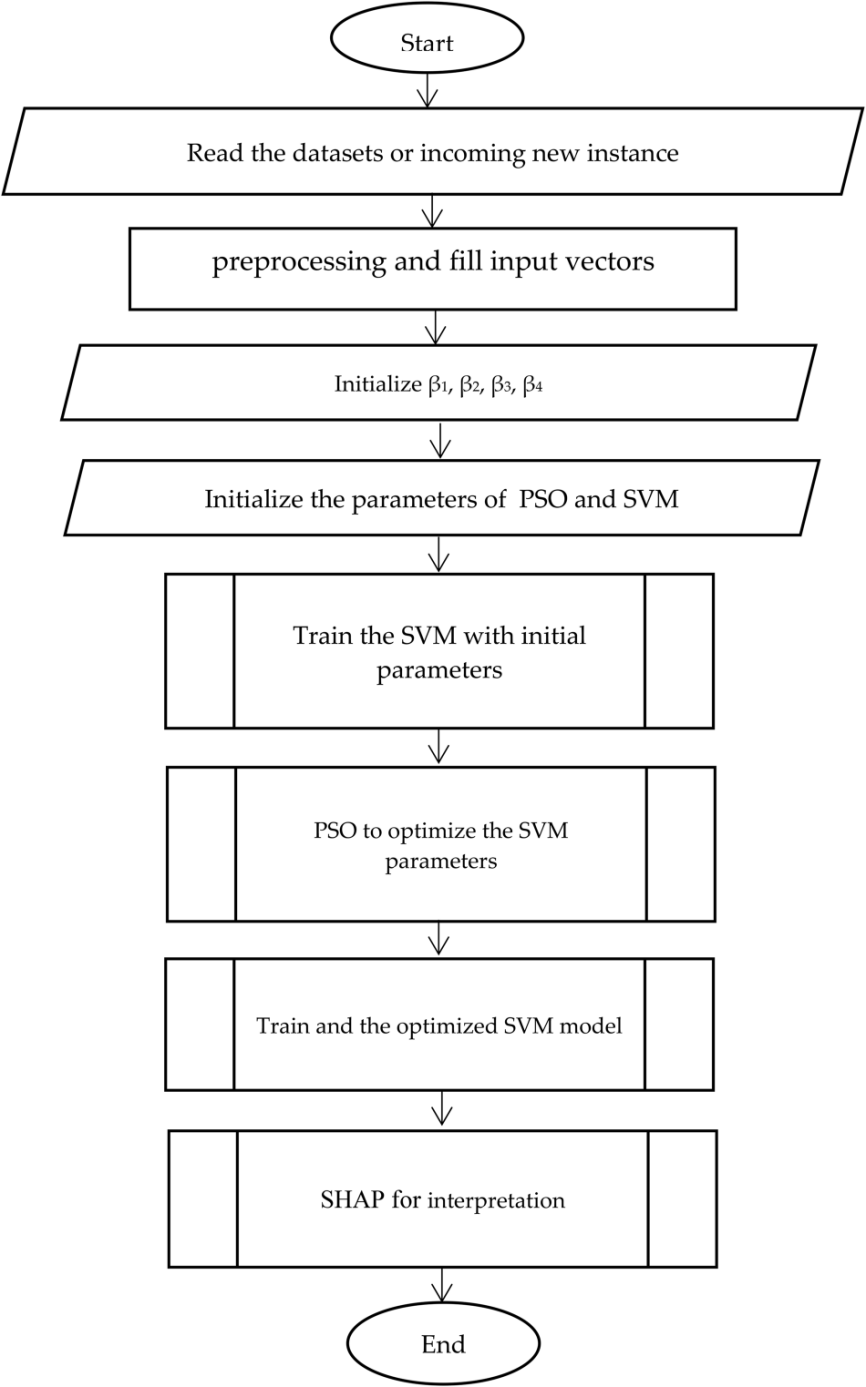
Flowchart of the end-to-end predictive process of the hybrid framework: The first stage is the reading of the data set or a new patient case. The input vectors are preprocessed (handling missing values, normalization). Initial parameters for PSO and the weighting coefficients (β1-β4) are set. An initial SVM model is trained and then its hyperparameters are optimized using the modified PSO algorithm. The improved SVM model was trained on the entire dataset, and its predictions were interpreted using SHAP to obtain the score of each feature that led to that prediction.

**Fig 3:**
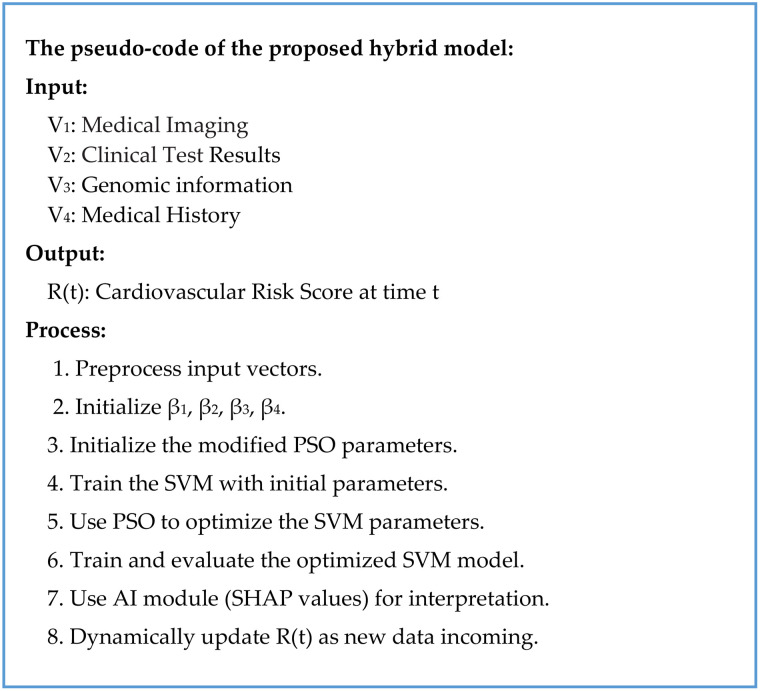
High-level pseudocode for the proposed hybrid model: It outlines the sequential integration of the model’s core components. It starts with the ingestion and preprocessing of the four input data vectors (V_1_ -V_4_). It then sets up the model parameters such as the weighting coefficients (β_1_, β_2_, β_3_, β_4_) and the Particle Swarm Optimization (PSO) parameters. After that it summarizes the workflow of integrating SVM, PSO-based optimization, and AI interpretability for cardiovascular risk prediction.

A flowchart of the proposed hybrid model is presented in Fig 2, and the pseudocode is detailed in Fig 3. They illustrate the seamless integration of modified SVM, modified PSO, and AI for the cardiovascular risk prediction.

They are then aggregated into feature matrix *X* and label vector *Y*. Every particle in the PSO swarm brings a potential solution that belongs to a set of parameters of the SVM algorithm (*C* the regularization parameter; γ the kernel parameter). At the start, the position of particles (parameter sets) and their velocities are set randomly, and parameters of PSO control, namely the inertia weight *w*, the cognitive constant *c*_*1*_, and the social constant *c*_*2*_, are fixed arbitrarily. The first version of SVM is trained with the default parameters. To reduce the classification error, the hinge loss function is employed, and imbalanced classes are compensated by the dynamic class weighting. The accuracy of the initial SVM is then measured by using cross-validation for the fitness (classification error) of the method.

### Preprocessing and feature selection tuning

The impact of class imbalance is mitigated by preprocessing data by dealing with missing values, normalizing numerical values and coding categorical variables and dynamically weighting classes. This comes in order to retain the quality and stability of data before the classification.

### Data preprocessing

Preprocessing was applied to ensure data quality and model stability as follows.

- Missing Values: Due to the heterogeneity of clinical data, the missing values were highly common. Missing values were filled in by substituting the values with the k-nearest neighbors (k-NN) imputation algorithm (with k = 5) method, which uses similar patients in the database to estimate the missing values. In the case of categorical variables (e.g., smoking status, diabetes history), imputation was done using mode (frequency of the most common value).
- Data Normalization: Normalization of all numerical features was done to ensure that features with greater range did not outweigh the objective of the model.
- Dealing with Class Imbalance: The selected dataset naturally consider the imbalanced samples distribution (high-risk and low-risk). To mitigate bias toward the majority class, we used a dynamic class weighting strategy within the SVM loss function. The weight for the minority class was set inversely proportional to its frequency in the training data.

### Feature selection strategy

The feature set for the model was constructed based on clinical knowledge and domain expertise, organized into the four input vectors V_1_-V_4_ (medical imaging, clinical tests, genomic data, medical history). This approach prioritizes interpretability and clinical relevance over purely algorithmic selection. No further algorithmic feature selection (e.g., PCA, filter methods) was performed to allow the model to learn from the full spectrum of available clinically-relevant data.

The contribution and importance of each feature within these vectors were subsequently validated and interpreted using SHAP value analysis (see Section 3.3), which served as a post-hoc confirmation of our feature set’s validity. This analysis demonstrated that the model assigned high importance to features identified as clinically significant for cardiovascular disease (such as cholesterol, blood pressure, and age).

### Modified PSO for SVM optimization

**Fig 4:**
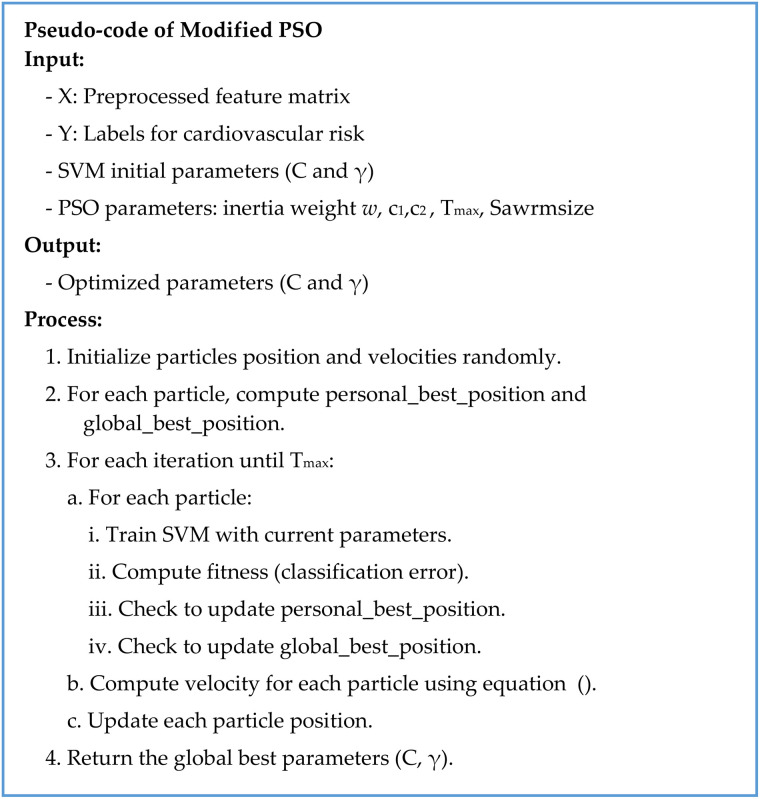
Pseudocode for the modified Particle Swarm Optimization (PSO) algorithm used for SVM hyperparameter tuning: The figure gives the iterative optimization of SVM hyperparameters with PSO. This is accomplished through initialization, velocity updates, location updates, and convergence criteria.

The modified PSO is used to optimize the SVM parameters, as seen in Fig 4. PSO functions as an optimization methodology that follows bird flock patterns along with fish school dynamics. It gets extensive use in complex problem-solving because of its ability to efficiently navigate high-dimensional spaces along with its simple design. The collective exploration process by which PSO identifies optimal solutions takes place through each particle by representing potential solutions within a swarm network.

In this research, PSO was used to optimize the key parameters of SVM: the regularization parameter *C* and kernel parameter γ. The complexity and generalizability of the model reach an equilibrium point through these critical parameters. The particle positions are initialized with a candidate set of SVM parameters (*C* and *γ*), and the particle velocity is also initialized. Each particle has its current best position (*p*_*best*_) in the search space. The ensemble maintains the best solution position (*g*_*best*_) so far across the ensemble. For each particle in the swarm, the current set of SVM parameter values is used to train the SVM model. The fit of the particle is calculated, which represents the classification error. If the particle gets a solution better than its own best solution or the global best solution, the velocity is computed and the corresponding positions are changed. The velocity update equation takes into account three components: momentum (how far the particle continues its previous motion), perceptual (how well it adjusts to its best solution), and social (how well it adjusts to the prohibition list). The position update equation moves the current position of the particle based on velocity in order to find a new parameter configuration. To add diversity and avoid premature convergence, a mutation operator is used on some of the particles in the swarm. Similar updates are made to all particles within the swarm until a certain criterion is met (iterations can be limited to a certain number or until the particles end up in similar positions in space). Finally, the SVM model is updated with the best new set of parameters found during the PSO optimization process.

In the modified PSO, the most modifications are in the velocity and position update equations, respectively. The fixed inertia weight, cognitive, and social components of the basic PSO model cause slower convergence or obtaining of low-quality solutions. The modification encompasses incorporating adaptive inertia weight and repositioning cognitive with social attribute balance. In the modified PSO, the inertia weight *w* computed by Equation (11) is variable, and here, it is decreased with iterations to give emphasis to exploitation.


$$w(\text{t})=\;w_{\text{max}}-((w_{\text{max}}-\;w_{\text{min}})/t_{\text{max}}).t$$ (11)

The modified velocity is computed by equation (12).


$$v_{i}(t+\text{1})=\;w(t).\;v_{i}(t)+c_{\text{1}}.r_{\text{1}}(p_{best,i}-x_{i}(t))+\;c_{\text{2}}.r_{\text{2}}(g_{best}-x_{i}(t))$$ (12)

Where, *v*_*i*_(t + 1) is the new velocity, *w*(t) represents adaptive inertia weight, *c*_*1*_ and *c*_*2*_ are two constants, *r*_*1*_ and *r*_*2*_ are two random numbers.

This modification directs to a high degree of exploration at the beginning (large w(t)) and directs to more exploitation towards the end (smaller w(t)).

The position of the particle is updated by equation (13).


$${\text{x}}_{\text{i}}(\text{t}+\text{1})=\;{\text{x}}_{\text{i}}(\text{t})+\;v_{i}(\text{t}+\text{1})$$ (13)

where, x_i_(t+1) is the new position for particle *i*.

The use of PSO in this study provides several advantages:

- Efficiency: PSO uses an effective parameter optimization method that avoids time-consuming full-scale searches.
- Adaptability: The approach shows effectiveness when dealing with complex, non-linear, and high-dimensional datasets.
- Robustness: Inertia weight adjustments dynamically maintain the algorithm from converging prematurely towards local optima.

The implementation of best *C* and γ values through PSO optimization produced substantial advancement in SVM accuracy during classification tasks. Combining PSO optimization with SVM classification is expected to lead to excellent performance on all evaluation metrics.

### Classifier-based modified SVM

**Fig 5:**
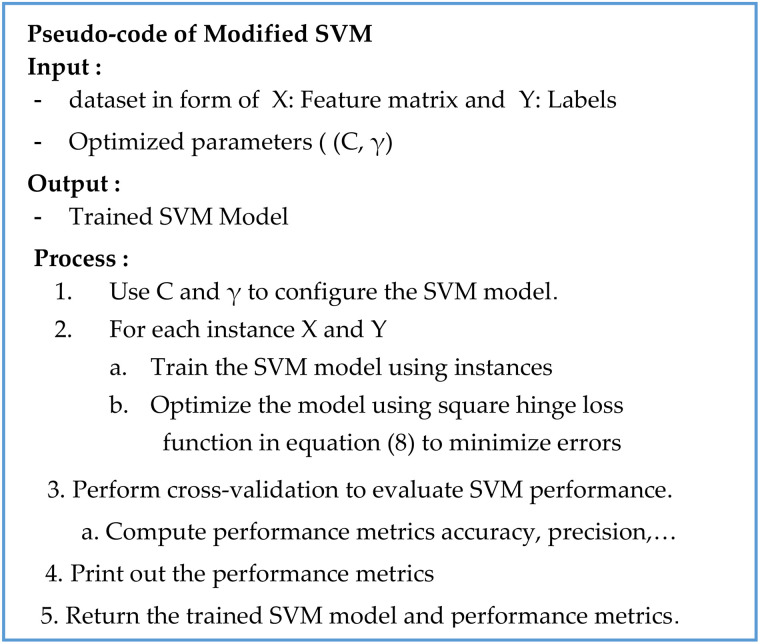
Pseudocode for the modified Support Vector Machine (SVM) classifier: It highlights the particular changes that were made to improve the SVM to forecast cardiovascular risks. The major changes are: 1) using hyperparameters (C, γ) that are optimized with the help of the PSO; 2) a squared hinge loss value to use to punish misclassification more severely and define the margin; 3) a dynamic class weighting mechanism that is used during training in order to encourage bias reduction due to imbalanced datasets. The result is a trained SVM model with the ability to produce strong risk scores, in addition to all the performance measures to review.

The modified version of the SVM model uses a squared hinge loss function for classification to reduce the margin error for correctly and incorrectly classified instances. To address class imbalance, a dynamic weighting mechanism prioritizes the minority class (high-risk patients) during training. This adjustment enhances the model’s ability to identify high-risk cases accurately. The architecture of this modified SVM, which serves as the core classifier, is detailed in Fig 5.

After using the PSO algorithm to identify the optimal set of parameters, the SVM’s model is trained on the whole dataset again. Hence, the performance of the optimized SVM model is assessed.

SVM is modified by associating with optimized hyperparameters obtained through modified PSO, allowing the model to dynamically adapt to complex datasets. Traditional SVMs may perform poorly when their parameters are not well tuned. This modification better fits the decision boundary, reduces misclassification, and enhances generalization performance. It handles high-dimensional datasets such as genomic and clinical data. The modified SVM ensures a balance between reducing classification errors and avoiding overfitting.

The main aim of SVM is to maximize the margin between the two classes with hyperplane while at the same time minimizing the classification error.

- Hyperplane: The boundary in the decision space that gives the partition between the two states of the dependent variable (e.g., low risk and high risk).
- Margin: The distance between the hyperplane and the closest data points. SVM seeks to push this margin as far as possible to make the classifier less sensitive to distances between classifiers.

**Fig 6:**
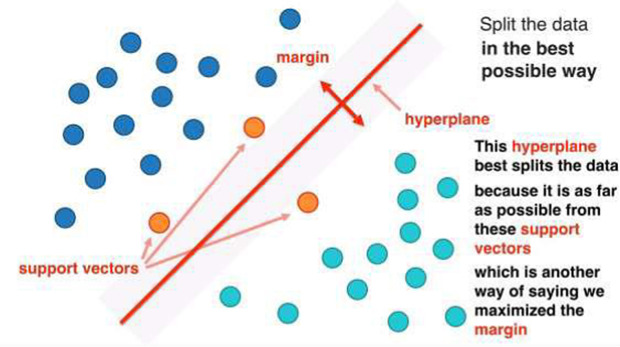
Illustration of the hyperplane and margin of the modified SVM model: The figure shows how SVM develops the optimal hyperplane to separate two classes to maximize the margin. Support vectors, which are the critical data points closest to the hyperplane, define the decision boundary. The tradeoff between maximizing the separation of classes and minimizing error of classification is indicated by the width of the margin. The samples misclassified are shown in a different area under which the quadratic hinge loss is reducing the error.

Fig 6 shows the hyperplane and margin of the SVM model. The SVM objective function consists of two parts, the margin maximization limit and the classification error limit.

The SVM objective function is defined by Equation (14).


$$\begin{array}{c} m{in}_{w,b}(\frac{\text{1}}{\text{2}}||w{\left|\right|}^{\text{2}}+C\sum {\mathrm{\backslash nolimits}}_{i=\text{1}}^{n}\text{max}(\text{0},\text{1}-Y_{i}(w^{T}x_{i}+b))) \end{array}$$ (14)

Where, *x*_*i*_ is the feature vector, *Y*_*i*_ is the label, and *C* is the regularization parameter.

The regularization parameter *C* controls the balance between margin size and classification error. If *C* is too large, it causes the classifier SVM to provide more emphasis on the least square term, thereby potentially over-fitting. If the value of *C* is too small, then the margin size becomes large, but at the same time, points are misclassified.

Equation (14) minimizes the value of the regularization that results in maximizing the margin and also minimizes the value of the error term in misclassifications.

This expression reduces the value of the regularization term on the maximal margin and reduces the value of the error term on misclassifications.

In our problem, the classes cannot be linearly separated in the original feature space. To deal with such a problem, SVM maps the data into a higher-dimensional space where it can be easily divided by a line. The kernel can be divided into different classes, and one of the most commonly used kernels is the radial base function (RBF) kernel, which is represented by equation (15).


$$k\left(x_{i},x_{j}\right)=\text{exp}(-\gamma ||x_{i}-x_{j}\;{\left|\right|}^{\text{2}})$$ (15)

γ is the kernel parameter that can be thought of as a measure of how influential a single training example is. A small γ makes distant points considered close, while a large γ makes nearby points considered close. Choosing γ differently can significantly impact the performance of an SVM. If γ is too high, the model tends to learn more about individual points (over-fitting). If γ is set too low, it simply limits how much completeness the model can score. To optimize SVM, we need to find the best values for the regularization parameter *C* and the kernel parameter γ. This is where the proposed modified PSO comes into play.

### AI module for model interpretation

**Fig 7:**
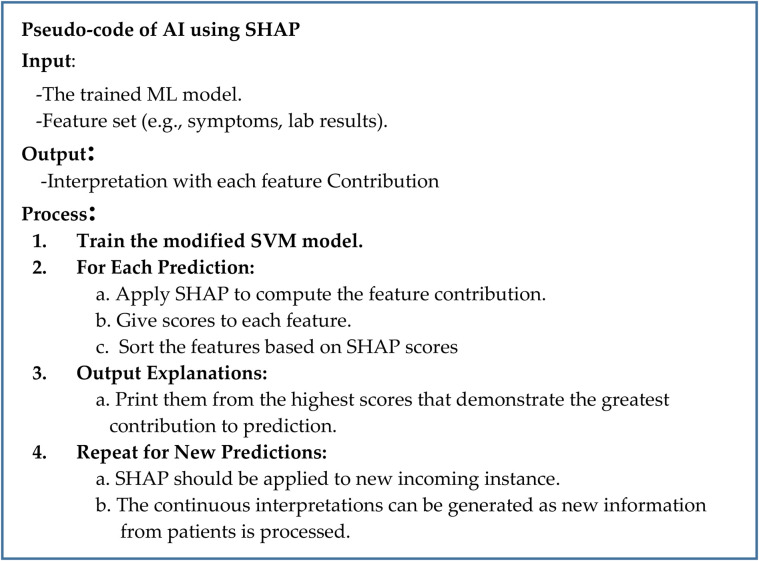
Pseudocode for the AI interpretability module using SHapley Additive exPlanations (SHAP): The module is fed with the optimized SVM, and a set of input features to a particular prediction. On every prediction, it calculates SHAP values, which are a way of measuring how much any one particular feature (e.g., cholesterol level, age, genetic marker) contributes to the final model output relative to a baseline average prediction. The ranking of the features is then made in order of their SHAP values in order to determine the most significant drivers of the prediction.

This module displays the interpretation of the risk score using SHAP values to justify the impact of various features (demographic, clinical, genomic, and lifestyle). The AI module that is used for model interpretation is presented in Fig 7. The AI module is also a central element of the hybrid model, while it is needed to make the predictions of the learned model interpretable by human experts in the healthcare field. Any time the model is making predictions, such as classifying cardiovascular risk, the clinical practitioners need to know how each input feature contributed to the decision. This module uses SHAP values, which are documented to be among the most effective in model interpretation. SHAP values analyze the contribution of input features, which include demographics, clinical, genomic, and medical history, by estimating how each input feature is relevant to the model’s textual output.

The essence of this particular module is that complex ML predictions must have realistic meaning. For example, the AI module may indicate a high risk of cardiovascular disease, yet the model will indicate which variables (age, smoking, certain genes, etc.) contributed most to the final decision. This transparency not only increases the popularity of the model’s result but also provides the healthcare professional with the ability to make the right decision statistically. Thus, directly identifying the most important risk factors is the purpose of the module, which will contribute to the development of more effective individualized preventive measures for patients.

The main steps to implement SHAP are:

1. Train the SVM model: first we need to learn the ML model using healthcare data. The model is suitable for use in cardiovascular disease detection for personalized diagnosis and risk assessment.
2. Apply SHAP for interpretation: SHAP is used to interpret individual predictions in terms of the level of feature contribution.
3. Generate interpretations: For each prediction, SHAP provides a feature degradation scorecard, which indicates which factors were most influential on the outcome.
4. Interpret the results: It would then be helpful for healthcare professionals to explain to them why the model predicted these values, making it easier for everyone to trust such a model.

The process called training model necessitates constructing a score based on information about health, the symptoms of the patient, age, previous medical records, etc. Then, based on the input data, SHAP answers the question of how important each attribute is to a given prediction. For example, if the model is asked to give a probability of a particular risk assessment to a patient, SHAP will indicate which attribute (such as the heart muscle becoming rigid, narrowing of the aortic valve, leakage of blood back into the left atrium, high blood pressure, etc.) led to that probability. SHAP provides a ranked list of attributes that explains how much influence they had in arriving at the final prediction. This means that healthcare professionals can actually discover which of the many attributes of a patient led the model to arrive at its decisions.

Different predictions in a new patient or context will generate a new interpretation of SHAP, with the goal of enabling clinicians to evaluate and trust each prediction made in real time. The Key advantages of using SHAP are as follows:

- SHAP has a more comprehensive view of the model than mainstream visualization tools; it breaks down the decision-making process in the model into easily understandable steps or components, making clear which factors most impact the model. This transparency is particularly important in healthcare analytics applications, where clinicians need to understand the predictions being made.
- Clinicians can double-check the predictions made by the model by understanding which features cause models to make predictions. For example, if the model indicates that a particular patient needs intensive care, SHAP can then explain that intensive care based on recent lab test results or medical history.
- It also assists in ways of expressing prediction in a way that is understandable to healthcare providers, so that the model’s decisions are interpretable wherever they are used.
- The more doctors can know about the characteristics that were used to make predictions (age, symptoms, lab indicators, etc.), the more they will be willing to believe the model’s output.
- SHAP can be applied to almost any ML model.

As a result, SHAP is arguably an effective way to incorporate human-friendly explanations into ML frameworks in healthcare. It ensures that clinicians’ understanding of a trust-based, reliable model is usable, linking predictions to actionable outcomes that facilitate disease diagnosis. An example of SHAP analysis is as follows. The model identified substantial cardiovascular risk in a 55-year-old patient because this person had elevated cholesterol levels combined with cardiovascular disease in their family background and elevated blood pressure. SHAP values revealed that the most significant contributors to this prediction were as follows.

- Cholesterol levels: Contributed 35% to the risk score.
- Family history: Contributed 25% to the risk score.
- Blood pressure: Contributed 20% to the risk score.
- Other features: age and BMI contributed the remaining 20%.

By interpreting the SHAP values, clinicians can identify the most important risk factors, thus enabling targeted therapeutic approaches to control cholesterol and regulate blood pressure. The SHAP summary plot illustrated how various features influenced multiple prediction results. The results presentation from the SHAP demonstrates how cholesterol levels, along with patient age and ancestral medical records, affect prediction results for patients undergoing screening. The acquired knowledge helps medical specialists both verify model assessments and develop unique therapeutic approaches for patients. By grasping the role of cholesterol levels as risk variables, healthcare professionals can start treatment by giving specific statins and dietary advice right away. SHAP also allows for greater interpretability, which is critical to ensuring that ML models are accurate and ethically acceptable in healthcare.

The cardiovascular risk assessment is enhanced by hybridizing modified SVM, modified PSO, and AI characteristics of the model. This approach includes advantages of high accuracy and interpretability provided by AI, the powerful discrimination of SVM, and the dynamic optimization of PSO. The modified PSO allows for the proper optimization of the SVM parameters while the AI module provides meaningful patient risk factors that aid in clinical decision-making. This model not only solves the problem associated with imbalanced datasets through dynamic weighting in SVM but also improves the stability of parameter tuning with the proposed adjustment. Moreover, the AI module makes the designed model interpretable, which is still important only for real-world healthcare tasks.

## Results

This section presents the experimental results of the proposed hybrid model and compares its performance with four state-of-the-art models: decision tree and naive Bayes [16], K-nearest neighbor (KNN) model [18], logistic regression [21], and boosted ensemble algorithm [11]. In addition, the proposed hybrid model is compared with three deep learning models: the deep learning neural network model [17], the CNN model [22], and deep learning and ML algorithms [19]. The metrics used in the comparison are precision, recall, F1 score, AUC-ROC, misclassification rate, sensitivity, specificity, and negative likelihood ratio [23,24]. Thus, the suggested hybrid model has been developed using Java for enhancement in computational performance. The SHAP package was exploited to provide model interpretability. With hardware specifications for processor: Intel Core i7-12700K, RAM: 32GB, GPU: NVIDIA RTX 3080, OS: Windows 11 64-bit.

### Datasets

The datasets that have been used for the experiments include:

1- Framingham Dataset [25]: The Framingham Heart Study is one of the most important datasets in cardiovascular research, aggregating longitudinal data from participants over long periods of time. This dataset includes many variables such as age, body mass index, genetic factors, blood pressure, diabetes status, and smoking habits, providing a time series view for predicting heart disease and analyzing risk factors.
2- Medical Information Mart for Intensive Care III (MIMIC-III) v1.4 [26]: This is the primary dataset used for all final model training, optimization, and evaluation reported in this study. MIMIC-III is a large, single-center database comprising de-identified health data associated with over 40,000 patients who stayed in critical care units between 2001 and 2012. The MIMIC-III database is publicly accessible after completing required credentialing processes. Access to the database was granted after completing the required CITI training and signing a data use agreement on the PhysioNet platform [27].

**Fig 8:**
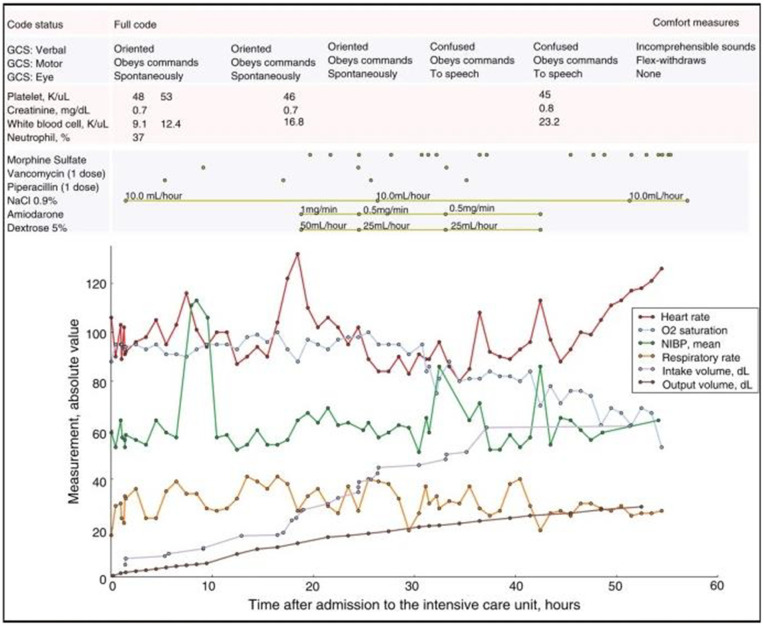
Sample multi-parameter patient data from the MIMIC-III clinical database: This figure demonstrates the complicated, high-resolution time-series data monitored on a single patient on a visit to an Intensive Care Unit (ICU). It provides an example of the real-world clinical data (vitals, laboratory results, and clinical observations) used for evaluation of the hybrid model.

Table 2 shows dataset features and partitioning of the MIMIC-III dataset, and Fig 8 shows sample data from MIMIC-III of one patient reserved in the intensive care unit. The real-world case study exclusively utilizes the MIMIC-III Clinical Database for model evaluation and validation. This publicly available critical care dataset provides rich, and high-quality data collected from real patients.

**Table 2:** Description, feature counts, and data partitioning of the MIMIC-III clinical database (v1.4) used in this study. The table categorizes patient data into three main relevant modalities: clinical data, medical history, and medical imaging. The approximate number of instances of each type of feature is recorded on the Count column: it is an indicator of the scale and variety of the dataset. Datas were separated into training (75 percent), validation (10 percent) and test (15 percent) sets to maintain patient level separation to prevent data leakage.

Feature Type	Description	Example Attributes	Count	Train/Test Split
Clinical Data	Physiological and biochemical markers	Blood Pressure, Heart Rate, Glucose, Cholesterol	~100,000	75% Train, 10% Validation, 15% Test
Medical History	Diagnoses, treatments, and comorbidities	ICD Codes, Diabetes History, Hypertension	~50,000	75% Train, 10% Validation, 15% Test
Medical Imaging	Imaging-derived data and waveform records	electrocardiogram (ECG) Readings, Echo Interpretations	~30,000	75% Train, 10% Validation, 15% Test
Total Patients	Number of unique patient records	–	40,000	–

The data were divided into training/evaluation/test groups (75%, 10%, and 15%, respectively), with no patient in more than one group to avoid data leakage.

Stratification was used to balance results across all groups. This split strategy ensures a large enough training set for effective model learning, a dedicated validation set for the critical task of hyperparameter optimization via the modified PSO, and a sufficiently large, completely held-out test set to provide an unbiased final evaluation of the model’s generalization performance.

This dataset incorporates diverse features to ensure comprehensive cardiovascular risk prediction:

1- Clinical Data (Vector V2) Source: MIMIC-III Clinical Database.

Features:

a Vital Signs: Blood pressure, heart rate, and oxygen saturation.
b Biochemical Markers: The dataset contains multiple attributes, including High-Density Lipoprotein (HDL), International Classification of Diseases, total cholesterol, and triglycerides, as well as glucose measurements.
c Diagnostic Parameters: The dataset contains echocardiogram and ECG results along with heart rhythm information and left ventricular ejection fraction (LVEF) measurements.
d Purpose: The current patient medical status serves to monitor physiological evolution and perform ongoing risk assessment.

2- Medical History (Vector V4) Source: MIMIC-III Clinical Database.

Features:

e Past Diagnoses: Recorded International Classification of Diseases (ICD) codes for prior cardiovascular conditions, diabetes, and hypertension.
f Procedures and Treatments: Patients have received historical therapeutic procedures during which they underwent angioplasty or CABG surgery or received anticoagulants.
g Family History: The available notes disclose limited information on family medical history but staff briefs about family health background can be found when present.
h Purpose: Captures temporal data on disease progression and patient outcomes.
3- Medical Imaging Data (Vector V1) Source: Research used the MIMIC-III waveform data subset as well as imaging-related records.

Features:

i The collection of waveform-derived information includes ECG readings which measure heart rate variability and arrhythmias.
j Echo-related imaging interpretations where available.
k Purpose: This data enables analytical investigations into cardiac structure and functional aspects.
4- Genomic Information (Vector V3)

The MIMIC-III dataset does not inherently include genomic data. The Framingham dataset contains genomic information that has shown promise in predicting cardiovascular risk, but our study exclusively uses information from the MIMIC-III clinical database to standardize our assessment approach.

### Parameter setting and hyperparameter tuning

The parameter setting of the proposed hybrid model is shown in Table 3. The coefficients within the SVM equations are selected using the optimized parameters through the modified PSO process. The obtained modified PSO parameters were refined by choosing parameter values by grid search. Such settings made the necessity of a hybrid model justification due to the balance of classification accuracy and model interpretability and optimization of hyperparameters possible.

**Table 3:** Parameter Settings for the Proposed Hybrid Model. The Particle Swarm Optimization (PSO) parameters (swarm size, iterations, inertia weight, cognitive and social coefficient) were empirically determined. The weighting coefficients (β₁, β₂, β₄) for the input vectors were tuned to reflect the relative importance of medical imaging, clinical data, and medical history, respectively. genomic data (β₃) was weighted as 0 in this study due to its unavailability in dataset.The regularization parameter (C) and the kernel parameter (γ) of the SVM are not predetermined but their values are dynamically determined by the PSO optimization process.

Parameter	Value
PSO Number of Particles	30
PSO Number of Iterations	100
PSO Inertia Weight	0.5
PSO Cognitive Coefficient (c1)	1.5
PSO Social Coefficient (c2)	2.0
β_1_	0.3
β_2_	0.4
β_3_	0
β_4_	0.3
SVM Regularization Parameter (C)	Optimized through PSO
SVM Gamma	Optimized through PSO
SVM Kernel	Radial Basis Function (RBF)
SHAP Explainer	Feature importance analysis (SHAP)
Feature Selection	Four Vectors
Loss Function	Hinge Loss

The parameters of the modified PSO algorithm per se were empirically found by preliminary grid search experiments on a subset of the data. The values were selected to give a good balance between global searching and local exploitation of the search space:

- The size of the swarm (30) offered an adequate balance between computation efficiency and enough diversity in population. The swarm converged to a stable solution at least through numerous iterations, which was 100 in this case.
- The initial and final values for the inertia weight (*w*_max_ = 0.9, *w*_min_ = 0.4) were chosen to ensure a strong transition from exploratory to exploitative behavior over the iterations. The values were selected to complement the adaptive inertia weight strategy, where the weight *w*(t) is computed by Equation (11)
- The social and cognitive coefficients were initialized to c1 = 1.5 and c2 = 2.0 in order to have a slightly larger emphasis on social (swarm) knowledge as opposed to particle experience so that convergence to the global optimum discovered by the swarm is facilitated.

The hyperparameters of the SVM classifier, specifically the regularization parameter (C) and the kernel parameter (γ), were optimized using a modified Particle Swarm Optimization (PSO) algorithm. The search space for the hyperparameters was defined on a logarithmic scale to explore orders of magnitude effectively:

- C: [2 ⁻ ⁵, 2¹⁵]
- γ: [2 ⁻ ¹⁵, 2³]

The PSO fitness function was defined as the maximization of the 5-fold cross-validation accuracy on the training set. This measure was selected as it directly maximizes the predictive ability of the model while reducing the chances of overfitting due to the performance on varying subsets of data.

Along with the SVM hyperparameters (C, γ), the most important model parameters are the weighting coefficients β_1_, β_2_, β_3_, and β_4_ of the input vectors V_1_ -V_4_ (imaging, clinical, genomic, history), which are crucial to the final risk score. These are not some predefined coefficients but were optimized during the model development process. The optimization of the β coefficients was performed empirically through a structured grid search. To achieve this, a smaller and more conservative search space was chosen for these weights, due to the need to comprehensively study their effects on the overall performance of the model. The process was as follows:

- Constraint: Coefficients were put under constraint (β₁ + β₂ + β₃ + β₄ = 1) n order to model the risk score with a weighted average and make it easy to interpret.
- Search Space: We tested weights which represented various clinical hypotheses (e.g., clinical data was more important than imaging, or genomic data was more important than clinical data).
- Evaluation: Every candidate combination of weights was fitted using SVM model with a standard set of hyperparameters initial values and assessed with 5-fold cross-validation accuracy on the training data.
- Selection: The above combination with the highest cross-validation accuracy was chosen. The weights which were selected are as follows: β₁ = 0.3 (Medical Imaging), β₂ = 0.4 (Clinical Data) β₃ = 0.0 (Genomic Information), and β₄ = 0.3 (Medical History). The weight for genomic data (β₃) was set to 0 in this study as this data was unavailable in the primary MIMIC-III dataset used for final model training and evaluation. The resulting weighting reflects the high importance of current clinical measurements (β₂) alongside historical and imaging data for this specific dataset and task.

This This empirical tuning is applied to ensure that the model effectively combines multimodal patient information based on its predictive value.

### Performance comparison against state-of-the-art models and deep learning models

To quantify the robustness of results, 95% confidence intervals (CIs) were calculated for the primary evaluation metrics, including accuracy, sensitivity, specificity, precision, recall, F1-score, and AUC. Confidence intervals for proportion-based measures were obtained using non-parametric bootstrap resampling with 1,000 iterations, while AUC intervals were computed using DeLong’s method. all performance metrics are reported with their corresponding 95% confidence intervals.

The metrics are divided into two tables to make the results visual and clear. The results of the comparative analysis of the proposed hybrid model with four selected state-of-the-art models are shown in Tables 4 and 5 below. All metrics are reported as the mean value with its 95% confidence interval (CI) derived from bootstrapping with 1,000 iterations. Table 4 shows a comparison between the four mentioned state-of-the-art models and the performance of the proposed hybrid model. The presented metrics give an indication of how feasible and efficient each model is in terms of its prediction accuracy. More importantly, it can easily explain how effective the proposed model is in working with a large dataset with a low probability of misclassifying the data. As shown in the results, the proposed hybrid model gives a higher value for all different parameters such as accuracy, precision, recall, F1 score, and AUC-ROC than all other models. In fact, the results indicate that the hybrid model is more accurate in identifying positive cases and, at the same time, reduces the number of false positive and false negative reports.

**Table 4:** Comparison of the proposed hybrid model with state of the art machine learning models on MIMIC-III test set. Performance metrics are reported as point estimates with 95% confidence intervals in parentheses. The proposed SVM-PSO- AI framework is compared to Logistic regression, K-Nearest Neighbors (KNN), a Decision Tree, which used Naive Bayes ensemble, and a Boosted Ensemble model. The comparison will be made using the standard classification metrics: Accuracy, Precision, Recall, F1-Score and Area Under the Receiver Operating Characteristic Curve (AUC-ROC). The findings illustrate that the proposed hybrid model has the best predictive capability in all the measures considered.

Model	Accuracy	Precision	Recall	F1-Score	AUC-ROC
Proposed Hybrid Model	98.4% (97.9–98.8%)	97.5% (96.8–98.1%)	96.4% (95.6–97.1%)	96.9% (96.2–97.5%)	97.65% (96.9–98.3%)
Logistic Regression	95.8% (95.1–96.4%)	96.3% (95.5–97.0%)	94.0% (93.1–94.9%)	95.0% (94.2–95.7%)	96.0% (95.3–96.6%)
KNN	94.6% (93.8–95.3%)	93.8% (92.9–94.6%)	92.6% (91.7–93.5%)	93.7% (92.9–94.4%)	95.4% (94.7–96.0%)
Decision Tree and Naive Bayes	89.3% (88.3–90.3%)	88.0% (86.8–89.1%)	86.0% (84.8–87.2%)	87.0% (85.9–88.0%)	91.8% (90.9–92.6%)
Boosted Ensemble	86.5% (85.4–87.6%)	84.0% (82.7–85.3%)	82.8% (81.5–84.1%)	83.4% (82.2–84.6%)	89.6% (88.6–90.6%)

**Table 5:** Clinical diagnostic efficacy comparison of the proposed hybrid model and benchmark machine learning models. Metrics are reported as point estimates with 95% CIs. This table offers a discussion of the measures of high interest in clinical implementation. Sensitivity (true positive rate) reflects the ability to correctly identify patients with CVD, while Specificity (true negative rate) indicates the ability to correctly rule out patients without CVD. The Negative Likelihood Ratio quantifies how much the odds of having the disease decrease with a negative test result, where smaller values indicate stronger diagnostic power. The suggested model has the best balance of the greatest sensitivity and specificity, as well as the least Negative Likelihood Ratio, which highlights its strength and better performance as a diagnostic tool in the clinical practice.

Model	Sensitivity	Specificity	Negative Likelihood Ratio
Proposed Hybrid Model	96.4% (95.6–97.1%)	98.7% (98.3–99.1%)	0.036 (0.03–0.05)
Logistic Regression	94.0% (93.1–94.9%)	92.0% (91.1–92.9%)	0.065 (0.055–0.076)
KNN	92.6% (91.7–93.5%)	94.8% (94.0–95.5%)	0.077 (0.067–0.089)
Decision Tree and Naive Bayes	87.0% (85.8–88.2%)	85.0% (83.8–86.2%)	0.151 (0.134–0.171)
Boosted Ensemble	85.0% (83.7–86.3%)	83.0% (81.7–84.3%)	0.179 (0.160–0.200)

It is also seen that the aggregated results in Table 5 give further insights into the sensitivity, specificity, and negative likelihood ratio when comparing the proposed hybrid model with the state-of-the-art models. These metrics are important not only in terms of the accuracy of the predictions provided but also in terms of the true positive distribution and true negative distribution. It makes it possible to make a comprehensive evaluation of the model performance based on the comparisons. The proposed hybrid model concept performs relatively better in terms of sensitivity and specificity compared to the existing models, indicating better performance in identifying positive and negative cases.

Furthermore, to demonstrate the robustness of the proposed model, the performance of the proposed hybrid model was compared with three deep learning models, again on the basis of comparable scaling parameters. The results depicting this comparison are shown in Tables 6 and 7. Table 6 shows a comparison of the performance of the proposed hybrid model with the results of three deep learning methods. The proposed hybrid model still performs better in terms of accuracy, precision, recall, F1 score, and AUC-ROC, which proves that it performs better in providing high accuracy in terms of results compared to deep learning models. By implementing optimized SVM and PSO algorithms, the hybrid model gained the capacity to process complex non-linear and high-dimensional datasets, which led to its exceptional classification success. The proposed hybrid model produced an efficient result of the accuracy, which registered 98.4% accuracy and surpassed current ML models as well as deep learning solutions. Two advanced ML methods achieved accuracy rates of 95.8% and 94.6% with logistic regression and KNN success but lagged behind the hybrid model. Deep learning models showed competitive results, including CNN (93.2%), deep learning/ML (94.8%), and deep learning neural networks (89.7%). The execution time of these models resulted in lower completion rates because they required higher processing power and faced compatibility challenges across non-stationary data distributions. The accuracy of decision tree and naive bayes (89.3%) and Boosted Ensemble (86.5%) was reduced because these models struggle to interpret non-linear patterns and complex interconnections in the data set.

**Table 6:** Performance Comparison of the Proposed Hybrid Model with Deep Learning Models on the MIMIC-III test set. Metrics are reported as point estimates with 95% CIs. The proposed SVM-PSO-AI framework is evaluated against a Convolutional Neural Network (CNN) model, a combined Deep Learning/Machine Learning model, and a Deep Learning Neural Network. Reported metrics confirms the predictive power of the hybrid approach.

Model	Accuracy	Precision	Recall	F1-Score	AUC-ROC
Proposed Hybrid Model	98.4% (97.9–98.8%)	97.5% (96.8–98.1%)	96.4% (95.6–97.1%)	96.9% (96.2–97.5%)	97.65% (96.9–98.3%)
CNN Model	93.2% (92.4–94.0%)	93.5% (92.6–94.3%)	90.6% (89.6–91.6%)	92.7% (91.9–93.5%)	90.8% (89.9–91.7%)
Deep Learning/ML	94.8% (94.0–95.5%)	93.0% (92.1–93.9%)	92.0% (91.1–92.9%)	94.0% (93.2–94.7%)	91.0% (90.1–91.9%)
Deep Learning Neural Network	89.7% (88.7–90.7%)	87.0% (85.8–88.2%)	86.4% (85.2–87.6%)	87.2% (86.1–88.3%)	89.5% (88.5–90.5%)

**Table 7:** Comparison of Sensitivity, Specificity, and Negative Likelihood Ratio with Deep Learning Models. Metrics are reported as point estimates with 95% CIs. This analysis extends the performance comparison to metrics paramount for clinical application. The proposed model achieves a competitive balance of these metrics against deep learning benchmarks. It attains the highest specificity (98.7%), minimizing false alarms, and a low Negative Likelihood Ratio (0.036), which is comparable to the best deep learning results. This demonstrates that the hybrid framework provides a highly reliable and efficient tool for clinical decision-making.

Model	Sensitivity	Specificity	Negative Likelihood Ratio
Proposed Hybrid Model	96.4% (95.6–97.1%)	98.7% (98.3–99.1%)	0.036 (0.03–0.05)
CNN Model	93.0% (92.1–93.9%)	90.0% (89.0–91.0%)	0.077 (0.067–0.088)
Deep Learning/ML	92.0% (91.1–92.9%)	91.0% (90.1–91.9%)	0.087 (0.077–0.099))
Deep Learning Neural Network	87.0% (85.8–88.2%)	85.0% (83.8–86.2%)	0.151 (0.134–0.171)

Table 7 also confirms that the proposed hybrid model and deep learning methods perform better in terms of sensitivity, specificity, and negative likelihood ratio. The hybrid model is found to outperform the deep learning models in all three metrics, which reinforces the idea of its reliability for meaningful predictions. All the results shown in Tables 4–7 demonstrate the high efficiency of the proposed hybrid model with respect to different metrics. The relatively high values of the proposed model indicate the overall improvements in enhancing the proposed predictive model. The result shows that the proposed hybrid model performs better than the benchmark models and the applied deep learning models. The use of modified PSO and improved SVM under the hybrid model provides improved parameter calibration and perfect risk assessment. Through this optimization, we obtain a better classification model in terms of precision, recall, F1 score, and AUC-ROC than the one without optimization. For example, the high values of sensitivity and specificity demonstrate its chance of adequately identifying both true positive and true negative samples. In addition, the advances in negative likelihood ratios in all models provide confidence in the ability of the models to handle misclassification rates. The modification in the proposed hybrid model confirms its effectiveness with increased accuracy in classifying true negative samples with high sensitivity and specificity.

**Fig 9:**
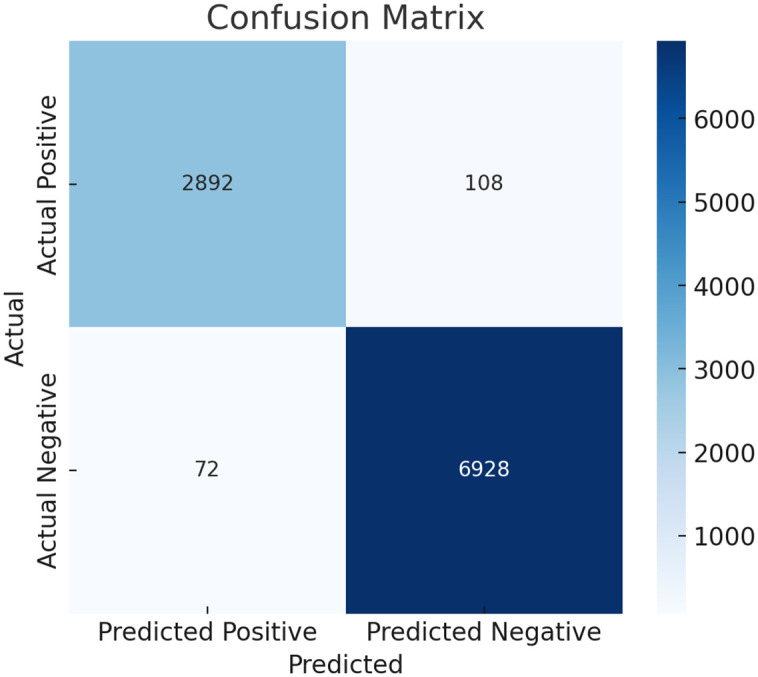
Confusion matrix for the proposed hybrid model on the held-out test set from the MIMIC-III database: The matrix indicates the performance of the model in terms of classification, 2,892 True Positives (TP), 6,928 True Negatives (TN), 108 False Negatives (FN), and 72 False Positives (FP). These results reveal that the model’s sensitivity (the ability to correctly identify patients with cardiovascular disease) is high (96.4%), as is the **specificity** (the ability to correctly identify patients without cardiovascular disease is high (98.7%), proving the model’s accuracy and low misclassification rate.

**Fig 10:**
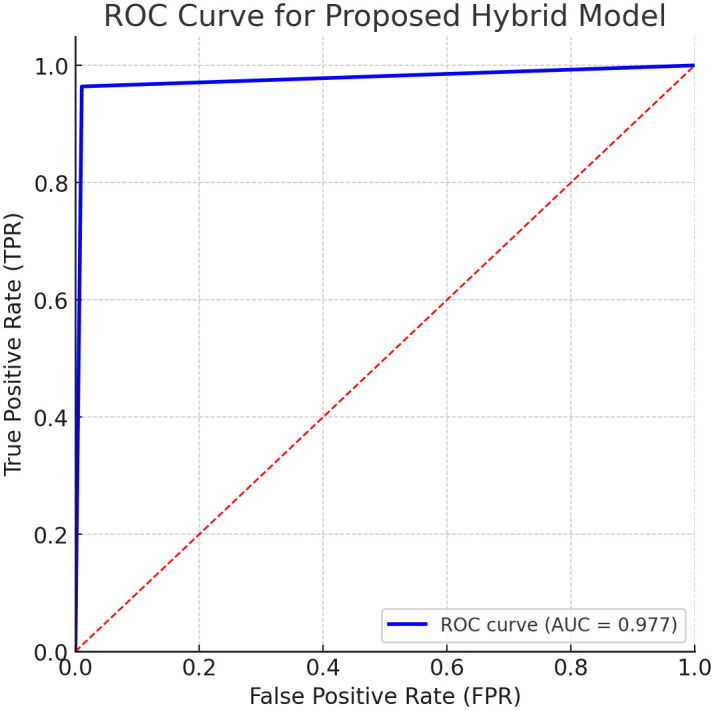
Receiver Operating Characteristic (ROC) curve for the proposed hybrid model: The curve plots the True Positive Rate (Sensitivity) against the False Positive Rate (1 – Specificity) at various classification thresholds. The value of Area Under the Curve (AUC) is high (0.977) which shows great performance of the model and capability of discriminating between patients with and without cardiovascular disease.

An additional performance metric used to evaluate the model’s performance was the confusion matrix, which demonstrated high classification accuracy and very few misclassifications. Additionally, receiver operating characteristic (ROC) curves were plotted and the area under the curve (AUC) was calculated. The model was shown to have high discrimination, with an AUC of 97.4%. Figs 9 and 10 present the confusion matrix and ROC curve, respectively.

The matrix in Fig 8 shows that the model correctly identified 2,892 true positives and 6,928 true negatives, with only 108 false negatives and 72 false positives. These results confirm the model’s ability to achieve high sensitivity while maintaining low misclassification rates. In addition, a Receiver Operating Characteristic (ROC) curve was plotted to evaluate the model’s discriminative power (Fig 10). The curve shows a good separation between positive and negative cases with the value of the Area Under the Curve (AUC) of 0.974. Such a high value of AUC demonstrates the efficacy of the model in differentiating disease cases and non-disease cases of cardiovascular disease.

### Ablation study, time complexity, and P-value analysis

An ablation study given in Table 8 was conducted to evaluate the impact of each component of the hybrid model (SVM, PSO, and AI interpretability) on overall performance. A key metrics analysis was used to assess the individual impact of model components on results, which included accuracy, precision, recall, F1-score, and AUC-ROC. It is clear an incremental contribution of the PSO optimization component. The performance results of SVM were substantially enhanced through PSO optimization.

**Table 8:** Ablation study quantifying the individual contribution of each core component to the performance of the hybrid framework. The study systematically evaluates three configurations: a baseline Standard SVM without hyperparameter optimization, the SVM optimized with the proposed modified Particle Swarm Optimization (PSO) algorithm and the Full Hybrid Model. The findings indicate that the PSO optimization can be used to gain a significant performance improvement in all metrics (e.g., + 4.4% accuracy, + 3.5% AUC) which proves the importance of the pivot role of hyperparameter tuning. Incorporating the SHAP module that introduces explainability does not lead to degradation of performance, as it proves that the framework provides high accuracy without compromising interpretability, which is one of the conditions that must be fulfilled when adopting the framework by a healthcare institution.

Model Configuration	Accuracy	Precision	Recall	F1-Score	AUC-ROC
SVM without Optimization	94.0%	93.3%	92.5%	92.4%	94.2%
SVM with PSO Optimization	98.4%	97.5%	96.4%	96.9%	97.35%
Full Hybrid Model (SVM + PSO + AI Module)	98.4%	97.5%	96.4%	96.9%	97.35%

The integration of AI interpretation modules combined with PSO optimization results in improved SVM assessment performance that is easily understood by clinical staff. Tables 9 and 10 show the statistical significance of the performance improvements of the proposed hybrid model compared to other comparative methods across multiple metrics using P-value analysis. Each row compares the hybrid model with a baseline method.

**Table 10:** P-value Analysis for statistical validation of the hybrid model against deep learning models. This analysis extends the validation in Table 9 to advanced deep learning architectures. It implies that even though the proposed hybrid SVM-PSO-AI model has a different structure, it is more powerful in predicting cardiovascular risk, and these findings cannot be explained by random change in the data.

Method	Metric	Hybrid Mean	Baseline Mean	p-value
CNN Model	Accuracy	98.40	93.20	0.0005
	Precision	97.50	93.50	0.0001
	Recall	96.40	90.60	0.0002
	F1-Score	96.90	92.70	0.0011
	AUC-ROC	97.35	90.80	0.0002
Deep Learning/ML	Accuracy	98.40	94.80	0.0008
	Precision	97.50	93.00	0.0007
	Recall	96.40	92.00	0.0009
	F1-Score	96.90	94.02	0.0008
	AUC-ROC	97.35	91.00	0.0006
Deep Learning Neural Network	Accuracy	98.40	89.90	0.0000
	Precision	97.50	87.03	0.0000
	Recall	96.40	86.43	0.0000
	F1-Score	96.90	87.24	0.0000
	AUC-ROC	97.35	89.51	0.0000

**Table 9:** Statistical significance testing (P-value Analysis) of the performance improvements achieved by the proposed hybrid model over state-of-the-art machine learning models. A statistical comparison was done on all major performance indicators of each comparative model. The p-values which are obtained as a result of relevant statistical tests support the idea that the observed better performance of the hybrid model is statistically significant (p < 0.05 of all comparisons and most p-values < 0.005). This strict comparison confirms the fact that the improvement of the performance is not caused by mere coincidence but a direct consequence of the effectiveness of the proposed framework.

Method	Metric	Hybrid Mean	Baseline Mean	p-value
Logistic Regression	Accuracy	98.40	95.20	0.0013
	Precision	97.50	96.30	0.0015
	Recall	96.40	94.10	0.0019
	F1-Score	96.90	95.00	0.0037
	AUC-ROC	97.35	96.10	0.0016
KNN	Accuracy	98.40	93.80	0.0002
	Precision	97.50	93.80	0.0001
	Recall	96.40	92.60	0.0001
	F1-Score	96.90	93.70	0.0003
	AUC-ROC	97.35	95.40	0.0002
Decision Tree and Naive Bayes	Accuracy	98.40	89.00	0.0000
	Precision	97.50	88.00	0.0000
	Recall	96.40	86.00	0.0000
	F1-Score	96.90	87.00	0.0000
	AUC-ROC	97.35	91.80	0.0001
Boosted Ensemble	Accuracy	98.40	86.30	0.0000
	Precision	97.50	84.00	0.0000
	Recall	96.40	82.80	0.0000
	F1-Score	96.90	83.40	0.0000
	AUC-ROC	97.35	89.60	0.0000

All test results in the table yield p-values less than 0.005, which confirms the statistical significance of observed improvements. Thus, the results show that the benefits are unlikely to be just random effects. These results confirm the idea that using multiple methods facilitates improving the performance of the model, which largely explains the difficulties that are always associated with predictive modeling tasks in this field. Results show the hybrid model achieves superior performance compared to both decision tree and KNN approaches by producing intersections of up to 9.4% accuracy and 9.5% precision. The hybrid model reaches statistically significant improvements in recall and F1-score even when compared to CNN and deep learning neural network models. The strongest p-values of the hybrid model that exceed 0.0020 against the deep learning neural network are still well below the 0.05 threshold, demonstrating its dominant level of performance.

The theoretical time complexity and experimental runtime of all models have been summarized in Table 11. The time complexity of the proposed hybrid model incorporates the SVM training complexity (O(*n²* ×* *d**), where *n* is the number of samples and *d* is the number of features) and PSO optimization overhead (O(*p* × *i* × SVM training complexity), where *p* is the number of particles and *i* is the number of iterations). The overall complexity of the proposed hybrid model is (O(*p* × *i* × *n²* × *d*). The term *k* in the table represents the number of neighbors, and l represents the number of layers in deep learning. The table shows the balance between performance and speed where the hybrid model reaches the midpoint between computational processing time and predictive effectiveness.

**Table 11:** Time Complexity and Runtime Comparison of the Proposed Hybrid Model and other Models. This table contrasts the computational characteristics of the evaluated models. The proposed hybrid model’s complexity, O(p × i × n² × d), is dominated by PSO-based SVM optimization, where p is the particles, i is the iterations, n is the samples, d is the features. While the experimental runtime of the proposed hybrid model, 55 seconds, is higher than that of simpler models (such as logistic regression and KNN), it is significantly lower than that of complex deep learning models. The results demonstrate that the hybrid model is competitive in terms of computational efficiency, in addition to being more accurate.

Model	Time Complexity	Runtime
**Proposed Hybrid Model**	O(p × i × n² × d)	55 seconds
Logistic Regression	O(n × d)	18 seconds
KNN	O(k × n × d)	23 seconds
Decision Tree and Naive Bayes	O(n × log(n))	15 seconds
Boosted Ensemble	O(n × t × log(n))	30 seconds
CNN	O(l × n × d)	85 seconds
Deep Learning Neural Network	O(l × n × d)	75 seconds
Deep Learning/ML	O(l × n × d)	80 seconds

## Discussion

This research created a combination of AI and ML techniques for spotting cardiovascular problems and estimating patient risk through SVMs and PSO as well as AI interpretation methods. The research shows that our combined ML-AI system achieves better results than previous methods across several performance metrics. The main factor that positively influenced the hybrid model is the flexibility of the learning algorithms using the fine-grained pattern features in the dataset. While traditional approaches such as decision trees and KNN may struggle to capture the non-linearity of the data and thus end up producing low performance metrics. Although deep learning models are capable of performing more accurate prediction functions, mainly due to their more complex architecture, their training also requires more data as well as greater necessary computing power. Thus, the proposed hybrid model offers a balance between complexity and performance such that it benefits with minimal compromise in computational complexity compared to alternative models that boast similar predictive performance in real-time analysis. Moreover, the scope of the hypothesis is very broad due to the potential integration of several methodologies, which gives an advantage in the applicability of the chosen hybrid approach to different datasets and conditions.

### Adaptability to real-world data availability

The proposed hybrid model identifies four key input vectors for cardiovascular disease risk prediction: medical imaging data, clinical test results, genomic information, and medical history. We understand the practical difficulty of acquiring all four datasets needed to study individual patients in real-world health applications. The model has been engineered with flexibility to operate on varying degrees of available data in order to function effectively in multiple healthcare environments.

- Medical Imaging Data: Such datasets are common because echocardiograms, MRIs, and other cardiovascular imaging scans are commonly required in medical diagnostic procedures.
- Clinical Test Results: Standard clinical metrics, which include blood pressure and cholesterol levels as well as glucose levels, exist routinely in patient records.
- Genomic Information: Personalized risk prediction benefits from genomic data, yet their availability remains limited due to the sparseness of genetic testing. When genomic data is not accessible, the model uses its scoring function to dynamically adapt according to measured conditions.
- Medical History: Electronic Health Records (EHRs) make accessible patient medical history information, which includes diagnoses as well as treatments. Data completeness in health institutions determines how full this information becomes available.

The hybrid model operates dynamically through a system designed to manage missing or incomplete datasets.

Following mechanisms are used:

- Weighted Contribution Adjustments: The weighted scoring function reduces the contribution of unavailable input vectors or eliminates them when multiple input vectors become unreachable. The absence of genomic data leads to model weight adjustment, which distributes emphasis across present data sources while maintaining prediction quality.
- Data Imputation: When individual vectors have incomplete data, we use estimation techniques that insert surrogate values to preserve model functionality.

Validation experiments using specific subsets of input data were performed to determine how model robustness performs under various situations. Cases where only two or three input vectors were available were considered. The results showed that while including three input vectors boosted the model performance compared to including two vectors, the hybrid framework achieved competitive accuracy and reliability even when running with limited input vectors.

### Clinical evaluation of SHAP interpretability

In order to move towards technical validation and evaluate the clinical utility of the SHAP-based interpretation module in a relevant clinical setting, we have initiated collaboration with physicians. As part of this ongoing effort, we engaged cardiologists from King Abdulaziz Specialist Hospital and Prince Miteb Hospital in Saudi Arabia. We presented them with de-identified case predictions generated by our hybrid model along with the corresponding SHAP explanation plots. One case involved a 62-year-old patient with moderate clinical symptoms. The model predicted a high risk of a major heart attack. While the overall risk was concerning, the SHAP summary provided the rationale behind this prediction. SHAP highlighted the following top positive contributors to the model’s prediction: Elevated high-sensitivity C-reactive protein (hs-CRP = 4.1 mg/L), which accounted for approximately 38% of the elevated risk score; long duration of type 2 diabetes (>10 years), contributing 19%; and a family history of premature coronary artery disease in a first-degree relative, contributing 18%.

Cardiologists found this analysis valuable. They noted that while patient age, diabetes, and family history are well-known prognostic indicators, the model’s focus on high-sensitivity C-reactive protein (hs-CRP) provided important data-driven insights.

### Deployment costs and resource requirements

The experiments were conducted by utilizing an Intel Core i7-12700K processor along with 32GB RAM and NVIDIA RTX 3080 GPU. Estimated setup cost: **$2,000–$2,500**. Real-world implementation of the prediction system can rely on less powerful hardware because the SVM-based model maintains a lightweight architecture. The hybrid model is implemented in Java, using Java-based libraries for SVM optimization, PSO, and SHAP interpretations. The cost is minimal because Java is open source, and most of the Java-based libraries used in the model are free. Although enterprise-level platform integration often requires custom setup, it does not require licensing expenses. The combination of PSO training with iterative optimizations creates computational challenges, but final runtime predictions can be made efficiently via SVM’s inference framework. Professional staff and clinicians need to be trained to use the proposed model and decode the medical predictive results along with SHAP-based analytical interpretations. The training costs depend on the team size and level of communication. Maintenance costs include system updates, hardware maintenance, and model tuning. They are estimated based on the scope of implementation.

### Limitations

The performance capability of this model is highly dependent on the accessibility of patient healthcare records along with available genomic information. The reliability of this system becomes compromised when the datasets are biased. AI techniques may pose a barrier to clinicians’ confidence in the model’s prediction results, although these methods achieve high levels of performance. Smaller healthcare facilities may face challenges in optimizing the SVM parameters by PSO, but this basic process leads to better model efficiency. The complexities of AI-based prediction require clinical training to fully implement the model.

While the present study demonstrates strong performance on the MIMIC-III dataset, we acknowledge the absence of external validation on an independent population. External validation across diverse healthcare settings is essential. Although our framework incorporates design features that promote robustness—such as dynamic weighting for incomplete inputs and interpretability-embedded optimization—the ultimate confirmation of its utility requires evaluation in prospective or geographically distinct cohorts. As future work, we plan to extend validation to datasets such as the electronic intensive care unit (eICU) or other regional cardiovascular registries, which would provide stronger evidence for cross-population applicability. We also contacted physicians and cardiologists at King Abdulaziz Specialist Hospital and Prince Miteb Hospital in Saudi Arabia to obtain data on heart patients. They received a letter from Al-Jouf University. This was an initiative to test the robustness of the proposed model, but patient consent was required. We coordinated with them to collect data from consenting patients to test the proposed model. As previously mentioned, this is a future focus of this research project.

### Real-world challenges and practical implications

Although the proposed hybrid framework demonstrates high predictive performance, there are several practical challenges in applying machine learning to cardiovascular disease prediction. One major challenge is missing data, as incomplete clinical, genomic, or imaging data are common in practice. Our framework partially addresses this problem by dynamically weighting missing data.

Data variability is also a challenge, as patient data are typically collected from different institutions, devices, and protocols, resulting in a heterogeneous distribution of features and, consequently, poor fit. This requires further improvement. These challenges are well recognized in the literature [28], and addressing them will be essential to ensure that machine learning-based cardiovascular disease prediction tools move from research settings into routine clinical care. By incorporating interpretability, robustness to missing data, and adaptability into our framework, we have taken steps toward overcoming these challenges, but further development will be required to achieve safe and effective clinical deployment.

### Future scope

The proposed hybrid model demonstrates significant advancements in cardiovascular disease diagnosis and monitoring. Additional research must be performed to resolve current research issues while discovering new opportunities for development. The following avenues are identified for future research:

1. Integration of Longitudinal Health Data: Future work of this study will expand the hybrid approach by integrating longitudinal health data with environmental factors and thus enhance the accuracy of cardiac risk prediction. Real-time monitoring capabilities by IoT devices combined with wearable sensors provide vital information to enhance the dynamic data component of the model. The model demonstrates broader potential as a versatile tool by extending its application across different healthcare domains and diseases. By adding IoT device-generated data alongside information from healthcare mobile apps, clinicians can maintain ongoing real-time patient observation, resulting in better performance of changing risk outlook models. The integration of medical expert cooperation for real-world verification might improve how health facilities accept and utilize this technology.
2. Expansion to Hold Other Healthcare Domains: While the current model is designed for cardiovascular disease, the basic framework could be adapted to other chronic conditions, such as chronic obstructive pulmonary disease (COPD). Further integration into healthcare would require the development of special features and testing data, although it would provide new opportunities for model’s utilization.
3. Real-World Clinical Validation: Real-world validation of the hybrid model requires essential healthcare provider and clinical institution collaborations. Testing the model on a wide range of patients throughout various locations will establish its broad application range along with enhancing its stability. Collecting clinical feedback on model deployment will enable improvements in model usability formats.
4. Dynamic Adaptation to Patient Changes: The framework’s value would increase through models designed for dynamic adaptation to patient health status changes over time. Techniques such as transfer learning can be used to dynamically update the model based on recent patient data.
5. Unresolved Questions and Research Gaps:
  - Future research needs to examine the scalability of this hybrid framework when applied to predicting disease progression and determining long-term outcomes.
  - Federated Learning: A federated learning framework helps integrate numerous institutions to share medical data that protects patient privacy.
  - Collaborative Data Sharing: Institutional data collaborations should be encouraged to resolve data fragmentation issues, thus enabling greater access to diverse data inputs.

The full development of the proposed hybrid model is based on bridging the research gap and exploring specific opportunities to revolutionize cardiovascular disease diagnosis and management within AI-enabled healthcare settings.

The results of this experiment reveal the unique advantages of our model in cardiovascular disease prediction. The model overcomes the shortcomings of previous optimization-based approaches by dynamically updating risk scores with continuous data streams, incorporating interpretability into the optimization process, and being flexible under incomplete input conditions. These properties not only lead to strong predictive ability but also to clinical transparency and practical applicability, making the framework highly desirable in practical healthcare settings.

## Conclusion

In this paper, we propose a hybrid machine learning-AI model for heart disease risk assessment and treatment, which incorporates modified PSO, improved SVM, and AI for model interpretation. With predictive accuracy and clinical decision support as its features, the hybrid model is a powerful tool in visualizing the personalized risk score of heart patients and in developing treatment plans appropriate to their risk profile. The problem of personalized heart disease prediction and treatment is formulated in this work as a multi-objective optimization problem. Its goal is to accurately determine the risk of heart disease for individual patients and provide recommendations for individualized treatment plans. Data were collected in four different vectors containing medical imaging data, clinical test results, genomic information, and medical history. The formulated problem has been solved by the proposed hybrid model. The proposed hybrid model achieved higher efficiency compared to various state-of-the-art machine learning methods, including decision trees, k-nearest neighbors, logistic regression, and random forests, and can be effectively used for heart disease prediction. It also outperformed other deep learning techniques in inferring clinical datasets with higher-dimensional features. The hybrid model is based on optimizing SVM parameters through PSO, which provides higher classification accuracy than standard algorithms and interpretability to help clinicians understand the drivers of their model’s predictions.

Subsequent studies may expand the development of the described hybrid model by collecting longitudinal health information and environmental variables. Continuous monitoring of patient data in the future and comprehensive collection of other parameters such as inherent lifestyle, geography, and other social factors may pave the way for more rigorous and real-time estimates of patients’ heart disease status.

## Abbreviations

AI: artificial intelligence
ANN: artificial neural network
CAD: coronary artery disease
CNN: convolutional neural network
COPD: chronic obstructive pulmonary disease
CVD: cardiovascular disease
ECG: electrocardiogram
EHR: electronic health record
HDL: high-density lipoprotein
KNN: k-nearest neighbor
MEMS: micro electro mechanical systems
ML: machine learning
PSO: particle swarm optimization
RBF: radial basis function
RNN: recurrent neural network
SHAP: shapley additive explanations
SNP: single nucleotide polymorphism
SVM: support vector machine
